# Considerations for the Development of a Substance-Related Care and Prevention Continuum Model

**DOI:** 10.3389/fpubh.2017.00180

**Published:** 2017-07-18

**Authors:** David C. Perlman, Ashly E. Jordan

**Affiliations:** ^1^Icahn School of Medicine at Mount Sinai, Mount Sinai Beth Israel, New York, NY, United States; ^2^Center for Drug Use and HIV Research, New York, NY, United States; ^3^Department of Epidemiology, School of Public Health, City University of New York, New York, NY, United States

**Keywords:** care continuum, substance use disorder, substance misuse, illicit drug use, substance use treatment, program evaluation, public health monitoring

## Abstract

There are significant gaps in the identification and engagement in care and prevention services of people who use illicit substances. Care continuum models have proven to be useful tools in the evaluation of care for HIV and other conditions; numerous issues in substance-related care and prevention resemble those identified in other continua models. Systems of care for substance misuse and substance use disorders (SUDs) can be viewed as consisting of a prevention and care continuum, reflecting incidence and prevalence of substance misuse and SUDs, screening and identification, medical and psychosocial evaluation for treatment, engagement in evidence-based treatment, treatment retention, relapse prevention, timeliness of step completion, and measures of overall and substance use-related specific morbidity and mortality. Care and prevention continuum models could potentially be applied at program, local, regional, state, and national levels. We discuss important lessons that can be drawn from applications of continuum models in other fields. The development and use of a substance-related care and prevention continuum may yield significant patient care, program evaluation and improvement, and population-level benefits.

## Introduction

Substance misuse and substance use disorders (SUDs) contribute to significant morbidity and mortality (1–3). In 2015, 8.5% (27 million people) of the general population reported current misuse of illicit or prescription drugs in the United States (4). Drug overdose deaths in the United States have nearly tripled between 1999 and 2015, accounting to 52,404 deaths in 2015 (5). The overall annual costs of illicit drug use and SUDs are $193 billion (4). In addition, there are significant gaps in the identification of people who use illicit substances, and who are screened for and engaged in needed treatment or prevention. An estimated 20 million people with SUDs do not receive treatment each year, and as many as 40 million people with substance misuse (with risky or harmful substance use patterns that do not meet SUD criteria) may also go untreated (6). Further, gaps in the implementation of substance use treatment have implications for other conditions; lower county-level access to buprenorphine is associated with county-level vulnerability to HIV epidemics (7).

The construct of “continua” has become an important tool in the evaluation and improvement of care for certain conditions (8–14). The construct is now part of domestic US public health evaluation systems for HIV and is a key component of the Joint United Nations Program on HIV/AIDS, and World Health Organization approaches to HIV global public health (15–17). It has provided valuable insights into the progress of individuals and populations through sequential steps of care, and into barriers to such progress (10, 13). The development and use of a substance-related care and prevention continuum may yield significant patient care, program evaluation and improvement, and public health-level benefits.

The continuum construct is also being increasingly used in other clinical and public health settings, such as in evaluations of care systems for other infections such as hepatitis C virus (HCV) (12, 18, 19), and non-communicable diseases such as diabetes and mental health disorders (20, 21). Estimates of the proportion of persons progressing through sequential continuum steps are useful for revealing net program- and population-level effectiveness of the specific aspects (e.g., screening, linkage to care, and treatment) of health-care systems that may contribute to suboptimal individual and public health outcomes. These estimates can inform interventions to improve identified gaps may have population-level health benefits. Yet, despite their increased use in some domains, continuum constructs have not been formally or consistently applied to a wide range of other conditions that might benefit from their use, such as substance misuse and SUDs. This is particularly notable since substance misuse and SUDs are frequently identified as barriers to progress through the continua steps for other conditions, such as for the HIV or HCV care continua (12, 22–24). This paper will explore considerations related to the development of a continuum model for substance misuse related prevention and treatment.

To some extent, a care continuum construct is implicit in much of the ongoing efforts for screening, brief intervention, and referral to treatment (SBIRT) implementation (25–27). Some have called for the formal development and application of continuum construct for selected substance-related settings, such as a tobacco continuum, and continuum models are being applied to alcohol use disorders and to behavioral health services for adolescent offenders (28–31). However, the application of a continuum construct to substance use, misuse, and SUD treatment and prevention more generally at program and population levels, and in particular as an explicit part of public health and health systems evaluations, may have unfulfilled potential (28–30, 32–35). We will use the term “substance-related prevention and treatment continuum” with the understanding that critical distinctions and decisions will need to be made.

## Discussion

There are important and valuable lessons that can be drawn from other applications of continuum constructs for the development of a substance-related treatment and prevention continuum that could serve as an important tool for program and public health evaluation of substance-related prevention and treatment efforts (8). Based on inferences drawn from review of successful continuum models applications [in HIV, tuberculosis (TB), and others] and their apparent limitations (8, 10, 16, 36, 37), we hypothesize that substance use can appropriately (and we suggest usefully) be viewed as consisting of a prevention and care continuum, likely including steps reflecting the incidence and prevalence of misuse and SUDs, screening/identification, medical/psychosocial evaluation for treatment, timeliness of step completion, engagement in evidence-based treatment, retention in treatment through to well-defined measures of treatment success, as well as degrees of engagement in evidence-based interventions to prevent relapse, and measures of overall and substance-related specific morbidity and mortality.

A continuum model for the care and prevention of substance misuse and SUDs might reasonably include a general population, a high-risk population, those with any substance use, risky use, or established SUDs and in fact the relevant population is one of the issues to be considered. Screening might focus on any use, on any recent use, on any measures of misuse, or any formal SUDs. There have been significant advances in the development of validated instruments and means of instrument delivery, including laptop-based self-report forms that facilitate implementation (25, 38–40). A valuable substance-related continuum model would apply some standardized definition of what constitutes screening (41, 42).

Screening, however, does not necessarily distinguish between incident (new misuse or SUDs) and prevalent (established, untreated or treated, misuse or SUDs) cases. Finding ways to distinguish between prevalent and incident cases in a substance-related continuum and to reflect measures of incidence in the models would improve their value as a public health tool. Additionally, as has been noted for HIV (16), screening is invaluable in identifying those in need of prevention, that is, those without misuse or SUD but who nonetheless have demonstrable risk and would benefit from prevention efforts. Further, given the often chronic relapsing nature of substance misuse and SUDs, appropriately measuring primary and relapse prevention efforts will be central to the development of sound substance-related continuum models (29, 33, 43, 44).

As with HIV and other conditions, issues of linking people from testing or screening steps to those of evaluation and treatment arise for substance misuse and SUDs (39, 45, 46). SBIRT and related interventions are important efforts to improve and expand screening and link identified people to care. Significant experience in HIV, HCV, and TB care suggests that processes of passive referral to treatment after screening yield inferior linkage outcomes compared to systems of active linkage (18, 47, 48). This issue may be particularly relevant to the evaluation of SBIRT implementation efforts, as studies have identified gaps in engagement in treatment post-referral, and the proportion successfully linked to further evaluation and treatment could be well monitored through appropriately developed substance-related continuum analyses (39, 45, 46).

While care continuum models can highlight gaps in the effective implementation of steps of care and prevention, they do not directly provide an understanding of the reasons for identified gaps. However, the development and use of care continuum models in conjunction with appropriate multilevel theoretic frameworks (49, 50), that can direct attention to the contributions of individual, social, and structural factors which impacts effective implementation make them useful in identifying and guiding efforts to address barriers and disparities.

As medical and psychosocial evaluations for substance misuse and SUDs treatment are not standardized and as specialist referral, diagnostic tests and treatment agents may require specific approvals, clinical evaluations may be individualized by providers influenced by unrecognized cognitive processes related to providers’ estimation of a patient’s resources, and real or perceived constraints imposed by patients, organizations, and insurers (51). These real and perceived constraints may pose barriers to care engagement and constitute a “stutter-step” in care provision and continuum progress (51). Therefore, it will be important to define continuum steps that reflect appropriate evaluations (to assess individuals and populations as “engaged in care”) and that can reveal where such procedures may pose barriers, and yet may be stable enough to allow analyses of changes in continuum progress over time.

For conditions such as HIV and HCV, “engaged in treatment” is taken to mean engaged in a care setting where a patient is evaluated for and offered specific pharmacotherapy (e.g., antiretroviral therapy for HIV or direct acting antivirals for HCV). For SUDs and substance misuse, another challenge is the diversity of treatment options available and in use for SUDs including various forms of pharmacotherapy, behavioral therapies, and combinations of pharmacotherapy and behavioral therapy, in outpatient, acute care, and residential settings. A substance use continuum would need to rely on appropriate assessments of evidence-based therapies in assessing proportions of persons with specific SUDs or substance misuse, as being “on therapy.” Guidelines and criteria such as those of the American Society for Addiction Medicine, Association for Medical Education and Research in Substance Abuse, and others may be useful both in categorizing different treatment types, and in assessment of proportions engaged in any treatment, as well as in patient-specific matched treatment (52–54).

Another key consideration would be defining measures of successful treatment. Again, for HIV and HCV, measures of viral suppression offer a clear, measurable biological marker of successful treatment response. In contrast, for SUDs and substance misuse, the heterogeneity of treatment options available result in a diversity of goal outcomes of successful treatment. Goal outcomes may vary from program completion, to successful continued engagement in treatment (e.g., retention in methadone maintenance) to cessation of substance use. These outcome measures may vary both by substance and by specific treatment modality. A broad measure of “successful treatment” could be considered as a summary measure for the various treatment modalities and substances under consideration, but use of validated outcomes for specific treatment modalities, i.e., outcomes that predict resolution of an SUD or relapse-free time, would be optimal.

One limitation of HIV continuum models as generally applied is their reliance on a surrogate marker of treatment response, i.e., viral load suppression as the key outcome. No direct biological marker exists for substance use treatment response, although negative toxicology tests and reports of sustained non-use are in some ways analogous. Nonetheless, as noted by nineteenth century epidemiologist William Farr “the death rate is a fact. Everything else is an inference” (55). A substance use continuum construct would include both measures of successful treatment and measures evaluating substance-related morbidity including transitions among illicit substances used and routes of use, measures of the resolution or absence of SUDs and substance misuse, and of rates of non-fatal overdoses, as well as all-cause and substance-related mortality.

We note that the term “continuum” has been used in a number of additional settings, some of which nonetheless have relevance for the models discussed here. One other use of the term “continuum” relates to instances of transitions across health-care system settings, e.g., individuals transitioned from inpatient to outpatient settings (56, 57). These aspects of care are clearly relevant to people who use drugs and who may transition between substance-related treatment in residential and outpatient settings. Other uses of the term refer to the continuity of care throughout the life course. This also may be particularly relevant for the substance-related continuum, since it is recognized that the prevalence of active substance misuse varies with age (increasing through adolescence and then decreasing over decades) and that rates of remission increase with age (43). It is therefore likely that proportions of people at different stages of a substance-related continuum would vary with the age distribution in that population and therefore, that comparisons of substance-related continua over time and between populations and settings may need to be age adjusted.

It would be critical to define the denominator most relevant to each specified continuum step, when appropriate reflecting a general population, or various specific high-risk populations, screened, those identified with risks for whom prevention may be appropriate, and those with identified misuse or SUDs in need of further evaluation or treatment. As with HIV and other continua models, the use of various population denominators will be important in addressing different questions (9). One limitation of currently employed HIV continuum models is that in depicting proportions who complete sequential steps does not reflect the time required to complete steps. Some representation of time may be depicted if the definition of that step includes time (e.g., “linked to care within a specified timeframe”). However, dichotomous measures of step completion can miss important variability in continuum progress (10, 36, 37). Finding ways to reflect the timeliness of progression from screening to evaluation to treatment would be important and valuable. It would be essential to identify appropriate data sources for each step (e.g., existing national surveys and reporting systems), relevant and valid measures of treatment success, standardized definitions of numerators and denominators, and standard methods to account for those who move or die and handling missing data.

Changes resulting from the Affordable Care Act (ACA) from 2010 leading to a greater emphasis on identification and management of SUDs within mainstream health-care systems, and the development and implementation of common screening and diagnostic information into electronic medical records (EMRs), have the potential to facilitate the engagement and retention of people in the substance use continuum models and to provide data sources for analyses of progress through continua models for SUDs and comorbid conditions (58, 59). However, for substance use issues, there may be unique challenges to the use of EMRs as datasets for continuum models given the requirements posed by confidentiality regulations including 42-CFR Part 2 (6). Redaction of SUD claims from Medicaid and Medicare datasets could significantly hinder use of electronic datasets as data sources for continuum analyses (60). Further, 42-CFR Part 2 intended to provide confidentiality protections to people with SUD may in fact adversely affect access to care for SUDs and associated comorbidities (50). Proposed changes in 42-CFR Part 2 might minimize these effects (61); however, the implications for substance use care, and its evaluation, of currently proposed changes in the ACA are unknown.

It would also be appropriate to reflect relationships between a substance-related continuum and continua for key comorbidities, for example, those for mental health and substance use-related infections (45, 62). Challenges to this include the number of potentially relevant comorbidities. Another challenge is that public health systems that collect information addressing one condition in one continuum model may not gather sufficiently detailed information about relevant aspects of interdigitating continua for multiple conditions either for reasons of simplicity, distinct institutional mandates, or particularly with respect substance-related data, issues of confidentiality.

Substance-related treatment systems have long had a predominant focus on acute episodes of misuse (48). The natural history of substance misuse and of SUDs is often a chronic one, involving cycles of relapse and recovery. Individuals may transition between periods of use, periods of treatment, and periods of recovery. Reflecting this chronic and cyclic nature of substance misuse and SUDs would be critical. A substance-related prevention and care continuum should depict the proportions of populations in each of these phases of relapse and recovery. As found for HIV continuum models (10), a cross-sectional prevalence continuum analysis (i.e., one that depicts the proportion of a population at any given step at a specific point in time) may not fully reflect the cyclical nature substance misuse and may overestimate retention in continuum models. Hence, a model that distinguishes between initial and subsequent treatment episodes and initial and relapsed periods of use, and which reflect the proportion of individuals progressing through sequential steps longitudinally (10, 36, 37) may more accurately reflect the state of substance-related diagnoses and care in a population.

Another key issue is whether to construct separate continua for the care and prevention of misuse of different illicit substances, or whether given the frequency of polysubstance misuse, to construct one overall substance-related prevention and care continuum and to use a diagnosis-based continuum model to conduct analyses among subpopulations who use different specific or different combinations of substances by any or selected routes of use (9).

One key challenge in developing and implementing a care and prevention continuum for evaluation of systems of substance-related care and prevention may stem from the fragmented nature of the system of care for substance misuse and SUDs. There are a plethora of involved entities such as schools, pediatric and adult medical practices, specialty SUD providers, criminal justice settings, and a patchwork of agencies that have few networks between them. However, the fragmentation of the substance use field not only poses challenges to continuum model development but also may itself contribute to gaps in progress through the substance-related continuum (63). Further, a substance use care continuum model would require clear definitions of the need for therapy, of what constitute evidenced-based treatments, of what constitute good outcomes, and measures of relapses. Substance-related continuum models may therefore be important ways to quantify these gaps and inform policy approaches to address them.

## Conclusion

Numerous issues in the delivery of substance-related treatment resemble those identified in the HIV, HCV, and TB continua constructs, including issues of underdiagnoses, gaps in linkages between screening and initial diagnosis and engagement in treatment, issues in treatment retention, adherence, and relapse, and the interdigitation of a substance care and prevention continuum with other continua. Clinical and public health systems addressing substance misuse and SUDs should be viewed and could be evaluated with use of care and prevention continuum models including steps reflecting incidence, screening/identification, medical/psychosocial evaluation for treatment, engagement in evidence-based treatment or primary prevention, retention in treatment through to specified measures of treatment success, timeliness of step completion, degrees of engagement in evidence-based interventions to prevent relapse, and measures of substance-related-specific morbidity and overall mortality.

These challenges in developing a feasible and informative substance-related care and prevention continuum are not insignificant. Nonetheless, addressing them and findings ways to represent outcomes of substance-related care and prevention as a continuum model may facilitate significant understandings of barriers to substance use care and prevention, domains in need of improvement, facilitate program evaluation, inform research and allocation of resources for care and prevention. Paralleling developments in other fields, use of a continuum model, which has proven to be both informative and intuitive to patients, providers, public health professionals, and policymakers, may also facilitate the further understanding and demarginalization of substance-related care and prevention. In conclusion, the development and use of a substance-related care and prevention continuum model may yield significant individual, programmatic, and public health benefit.

## Author Contributions

DP and AJ contributed to the design of the work; AJ drafted the first version of the manuscript and both DP and AJ revised it critically for important intellectual content and agreed to be accountable for the work.

## Disclaimer

The views expressed are the authors’ own and do not necessarily represent the views of the National Institutes of Health or the United States Government.

## Conflict of Interest Statement

The authors declare that the work was conducted in the absence of any commercial or financial relationships that could be construed as a potential conflict of interest.
